# Role of Statins in Oral and Maxillofacial Surgery: A Literature Review

**DOI:** 10.7759/cureus.69746

**Published:** 2024-09-19

**Authors:** Saloni J Kanabar, Deepankar Shukla, Nitin Bhola, Anchal Agarwal

**Affiliations:** 1 Oral and Maxillofacial Surgery, Sharad Pawar Dental College and Hospital, Wardha, IND

**Keywords:** bone regeneration, oral and maxillofacial surgery, oral health, periodontal disease, statins

## Abstract

In recent years, there has been a notable increase in interest in the use of statins in oral and maxillofacial surgery. The purpose of this literature review was to look into the effectiveness of statins in this area. Using a set of keywords, a thorough search of electronic databases was carried out, including PubMed, Scopus, Web of Science, Excerpta Medica database (EMBASE), and ProQuest. The papers considered were just those published in the English language between January 2012 and January 2024. Only human studies were taken into consideration; those involving animals were not. For the final analysis that assessed the use of statins in dentistry, a total of 30 papers were chosen. The designs, sample sizes, and materials employed in the experiments varied. According to the research, statins improve bone regeneration, have antiviral and antibacterial qualities, and work well as a therapeutic adjuvant for the treatment of periodontal disease. The analysis of the literature indicates that statins may be beneficial for treating periodontal disease, promoting bone regeneration, and improving oral health in the context of oral and maxillofacial surgery. Nevertheless, more investigation is required to completely comprehend the function of statins in this domain.

## Introduction and background

Low-density lipoproteins (LDL) are cholesterol particles associated with arteriosclerotic cardiovascular disorders. A class of drugs called statins primarily targets the liver's production of cholesterol [1]. They accomplish this by preventing 3-hydroxy-3-methylglutaryl coenzyme A (HMG-CoA), an essential stage in the production of cholesterol, from being converted to mevalonate [2]. Furthermore, studies have demonstrated that statins promote osteoblast activity and development by increasing the synthesis of bone morphogenetic protein-2, a chemical that aids in the transformation of cultured osteoblasts into bone-like tissue [3]. Statins may contribute to bone mending because of their capacity to promote bone growth [4].

A new era for statins began in 1976 with the release of lovastatin by Dr. Akira Endo and his colleagues at Sankyo Pharmaceutical (now a part of Daiichi Sankyo) and Merck & Co. [5]. The source of lovastatin was *Aspergillus terreus*. It was the first statin to lower cholesterol when it was approved by the U.S. Food and Drug Administration (FDA) in 1987 [6]. In the 1990s, the FDA also approved the use of simvastatin (Zocor) and pravastatin(Pravachol), increasing the number of statin alternatives available. Atorvastatin, also marketed as Lipitor, was first made available in the late 1990s and gained rapid popularity due to its strong cholesterol-lowering effects. Third-generation statins, including rosuvastatin (Crestor) and pitavastatin (Livalo), have been created with increased potency and fewer drug interactions [7-9].

An essential component of statins, HMG-CoA reductase inhibitors play a critical role in lowering cholesterol levels [10-11]. The effects of statins on absorption, distribution, metabolism, and excretion are partly attributed to the functional groups they contain, such as lactones and hydroxyl groups, among others. The potency, bioavailability, and pharmacological characteristics of various statins are influenced by the distinct side chains that are attached to their primary structures [12-15].

Statins are commonly recommended to treat familial hypercholesterolemia, manage dyslipidemia, prevent atherosclerosis, improve diabetic management, and avoid heart attacks and strokes [16]. Statins should not be used in certain situations, though, such as when co-occurring medications are being taken, when liver disease is active, when a woman is pregnant or nursing when there has been a history of statin-related muscular complaints, when severe kidney disease is present, and when known hypersensitivity responses have occurred [17].

Considering the intricate nature of oral and maxillofacial surgery and the necessity for practice that is supported by data, it is imperative to conduct a thorough examination of the existing literature in order to clarify the function of statins in this particular area of medicine. Hence, the objective of this literature review is to methodically analyze and condense the current data about the utility of statins in oral and maxillofacial surgery, investigating their possible advantages and consequences for surgical results.

## Review

Search strategy

The following keyword searches were used in the titles and abstracts of a methodical electronic search of the PubMed, Scopus, Web of Science, Excerpta Medica database (EMBASE), and ProQuest databases: “Oral" OR "Maxillofacial" OR "Surgery" OR "oral health" OR "oral diseases" OR "dental diseases" OR "oral cancer" AND "simvastatin" OR "statin" OR "rosuvastatin" OR "atorvastatin" OR "mevastatin" OR "lovastatin”.

Inclusion and exclusion criteria

Only English-language papers that were published between January 2012 and January 2024 were included in the search of the literature, to provide a more updated view of the efficacy of statins in oral and maxillofacial surgery. Only studies done on humans were included, and studies done on animals were excluded.

Results

**Figure 1:**
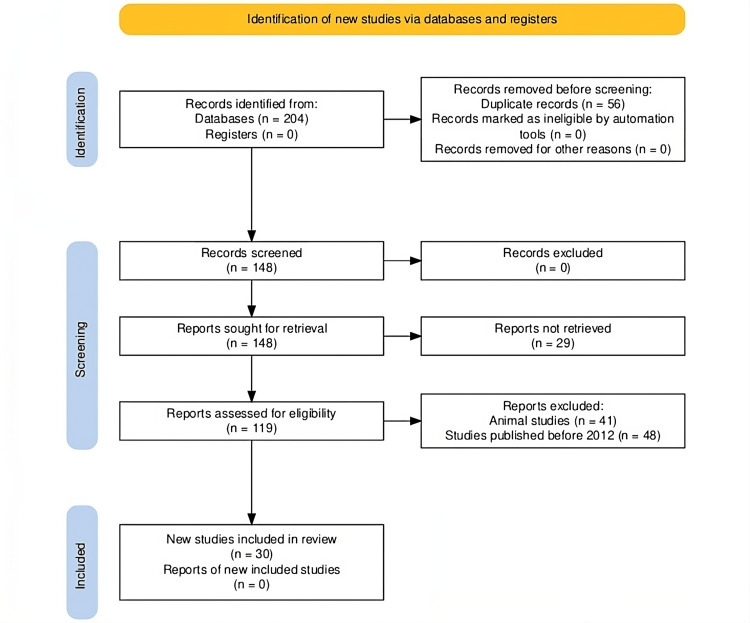
A PRISMA flowchart outlining the study selection process for this review PRISMA: Preferred Reporting Items for Systematic Reviews and Meta-Analyses

The initial stage of selecting research involved conducting a database search and obtaining 204 entries (Figure 1). After 56 duplicate entries were removed, 148 entries were evaluated. Subsequently, an effort was undertaken to acquire the complete text of every one of the 148 entries. Only 119 records remained for further analysis, though, as 29 reports were not retrievable. Forty-eight articles published before 2012 and 41 research studies involving animals were excluded when specific exclusion criteria were used. Ultimately, 30 papers that satisfied the inclusion requirements were picked for the comprehensive examination.

Table 1 presents the included studies and our interpretation of their findings. A variety of statins were utilized in these trials, which were published between 2008 and 2023. They included rosuvastatin, atorvastatin, and simvastatin. A variety of materials were used in these experiments; for instance, in some, statins were used in conjunction with gel foam or polypropylene membrane. These studies examined the effects of statins on bone augmentation and regeneration, as well as the potential uses of statins in the treatment of oral cancer, chronic periodontitis, and other dental health problems. The study's sample sizes ranged from one patient to over 48,000 participants. The research methods also varied; some were in vitro, while others were systematic reviews, retrospective cohort studies, and randomized controlled trials.

**Table 1:** Statins utilized across different studies till date in the field of oral and maxillofacial surgery N/A: not applicable; RCT: randomized controlled trial; PRF: platelet-rich fibrin; RCTs: randomized controlled trials; CBCT-PAI: cone beam computed tomography-periapical index; TCA: tricalcium phosphate; NSAIDs: non-steroidal anti-inflammatory drugs; SACC: salivary gland adenoid cystic carcinoma; TMJ: temporomandibular joint; SIM: simvastatin; LAO-G-gelatin: lactic acid oligomer-G-gelatin; PLGA/HA/b-TCP: poly (d, l-lactide-co-glycolide)/hydroxyapatite/beta-tricalcium phosphate; OSCC: oral squamous cell carcinoma; OAS: overall survival; RFS: recurrence-free survival; PDLCs: periodontal ligament cells

Study Name	Year	Material Used	Purpose	Parameters Assessed	Study Design	Sample Size	Results Observed	Overall Inference Drawn
Abdulrab et al. [18]	2019	1% Simvastatin mouthwash (20 mg tablet dissolved in distilled water)	COVID-19 (as antimicrobial, anti-viral, anti-inflammatory, antioxidant, immunomodulatory)	Anti-viral and anti-bacterial effects of statins	In vitro studies and meta-analysis	N/A	Statins showed promising anti-bacterial effects against periodontal pathogens and potential anti-viral effects against SARS-CoV-2	Topical statins may minimize the risk of SARS-CoV-2 transmission in dental settings, but clinical trials are needed
Abu Sheehah et al. [19]	2022	Simvastatin (10 mg) with normal saline	Bone augmentation and socket preservation in extraction socket	Bone regeneration, density, width, height	Comparative study	20 dental sockets	Significant increase in density, significant difference in width, no significant difference in height	Simvastatin induces bone formation, but cannot preserve alveolar bone height
Aljudaibi et al. [20]	2019	Simvastatin, rosuvastatin, and atorvastatin	Chronic periodontitis treatment	Efficacy of statins as an adjunct therapy for periodontal treatment	Systematic review and meta-analysis	15 studies	Statins, particularly simvastatin, improved periodontal parameters, including pocket depth and clinical attachment level gain, when used as adjunct therapy	Statins, especially simvastatin, may be beneficial as an adjunct therapy for periodontal treatment, but more research is needed
Ambrósio et al. [21]	2017	Simvastatin, Rrosuvastatin, atorvastatin	Adjuvant local delivery of statins	Adjunctive local delivery of statins, periodontal therapy	Systematic review and meta-analysis	10 RCTs	Local delivery of statins improved pocket depth and clinical attachment gain	Statins may be a promising adjunct to periodontal therapy
Bahrami-Hessari et al. [22]	2021	Systemic statins	Peri-implant bone loss	Peri-implantitis, bone loss, statin use	Retrospective cohort pilot study	60 exposed, 196 non-exposed	Statin use correlated with peri-implant bone loss	Statins may have a beneficial effect on peri-implant bone loss
Baliga et al. [23]	2018	Statin + PRF	Osteoradionecrosis of the lower jaw	Management of osteoradionecrosis with simvastatin and PRF	Case report	One patient	Remarkable healing of the non-healing socket with adequate gain in alveolar height.	A combination of simvastatin and PRF may be effective in managing Stage 1 osteoradionecrosis.
Cai et al. [24]	2018	Simvastatin (10, 20, 30, 40, 50 micromol/L	Human salivary	Proliferation, invasion, apoptosis, survivin expression	In vitro study	-	Simvastatin inhibited proliferation and invasion, induced apoptosis, and downregulated survivin expression	Simvastatin may be a novel target for SACC therapy
Cruz et al. [25]	2021	Simvastatin 1.2% with Polypropylene membrane	Healing of extraction socket	Dimensional changes, soft tissue healing, and pain perception in post-extraction sockets with simvastatin gel	Randomized controlled trial	26 patients	Simvastatin gel reduced dimensional changes, but not soft tissue healing and pain perception	Simvastatin gel is effective in reducing dimensional changes in post-extraction sockets
Degala et al. [26]	2018	Simvastatin (10 mg)	Regeneration of bone after surgical removal of Impacted third molars	Bone regeneration, pain, postoperative swelling, bone density	Randomized, split-mouth, single-blinded, single-center trial	30 patients	Significantly higher mean grey-level histographic values for study sockets at one, four, eight, and 12 weeks	Local application of simvastatin can stimulate and hasten osseous regeneration
Fu JH et al. [4]	2012	Different concentrations of statins	Implant healing and Osseointegration	Systemic medications, peri-implant bone healing	Literature review	-	Statins improve implant osseointegration, conflicting results for glucocorticoids and NSAIDs	Statins may improve peri-implant bone healing, but more research is needed
Gouda et al. [27]	2017	Beta-TCP and simvastatin (7.21 mg)	Sinus lift and osteoinductive capacity	Simvastatin, sinus augmentation, bone quality and quantity	Clinical trial	Six patients, eight sinus lift procedures	The simvastatin group showed higher newly formed bone, but a higher resorption rate	Simvastatin is safe and has promising osteoinductive capacity, but a larger sample size is needed
Gupta et al. [28]	2019	Simvastatin	Bone, soft tissue, and TMJ cartilage healing	Osteopromotive potential, soft tissue and TMJ cartilage healing properties of simvastatin	Systematic review	10 animal studies, six clinical studies	Simvastatin administration displays positive treatment outcomes in various oral therapies, including periodontal infection control and bone regeneration	Simvastatin is beneficial for the healing of oral bone and cartilage
Gupta S et al. [29]	2020	Simvastatin	Bone regeneration	Effectiveness of simvastatin, hydroxyapatite, and platelet-rich fibrin in bone regeneration of periapical defects	Randomized controlled trial	39 patients	Significant change in CBCT-PAI scores in the SIM group, indicating a faster rate of bone regeneration	Simvastatin is more effective in promoting bone regeneration compared to hydroxyapatite and platelet-rich fibrin
Harsha et al. [30]	2021	Simvastatin powder (10 mg) with gel foam	Third molar extraction socket for bone regeneration	Bone regeneration, density, pain, swelling	Randomized controlled trial	50 patients (100 extraction sockets)	Significant increase in bone density at first, fourth, eighth, and 12th weeks, reduced pain and swelling	Local application of simvastatin promotes and enhances bone formation in extraction sockets
Hayder et al. [31]	2021	Simvastatin powder (10 mg) with gel foam	Bone density after third molar removal	Bone regeneration, density	Prospective comparative randomized clinical study	24 patients (32 cases)	Significant increase in bone density three months postoperatively	Local application of simvastatin increases bone density in sockets of surgically extracted mandibular third molars
Jin et al. [32]	2021	Simvastatin	Bone regeneration	Molecular mechanisms of simvastatin on bone metabolism and angiogenesis, osteogenic differentiation, drug delivery systems	Review	-	Simvastatin promotes osteogenesis and angiogenesis, enhances bone regeneration	Simvastatin-loaded drug delivery systems have the potential for bone regeneration
Kabra et al. [33]	2023	Simvastatin	Different aspects of dental and oral health	Role of statins in oral health applications, including dentin regeneration, bone health, and wound healing	Review	-	Statins have promising effects on oral health, including dental pulp cells, chronic periodontitis, and alveolar bone loss	Statins, including simvastatin, have significant impact on enhancing oral health
Miyazawa et al. [34]	2015	A mixture of LAO- G-gelatin solution and Simvastatin solution	Odontoblastic differentiation	Odontoblastic differentiation, simvastatin, dental pulp stem cells	In vitro and in vivo study	-	Simvastatin enhanced odontoblastic differentiation and bone formation	Statins can enhance odontoblastic differentiation and bone formation
Noronha et al. [35]	2017	Poly (d, l-lactide-co-glycolide) with hydroxyapatite/b-TCP (PLGA/HA/b-TCP) scaffolds, 2.0% simvastatin scaffold	Extraction sockets of upper Third molars	Healing of maxillary third molars postextraction sockets with different ridge preservation techniques	Randomized controlled trial	26 sockets (13 patients)	Simvastatin-loaded scaffolds showed fewer clinical complications and graft loss	Simvastatin-loaded scaffolds are superior to others in ridge preservation
Park et al. [36]	2009	Simvastatin	Bone regeneration	Effects of simvastatin on bone formation, osteoblastic and osteoclastic activity, anti-inflammatory effects	Review	-	Simvastatin promotes bone formation, inhibits osteoclastic activity, has anti-inflammatory effects	Local application of simvastatin with carriers promotes bone formation
Saifi et al. [3]	2017	Simvastatin 10 mg mixed with gelatin sponge	Healing of extraction socket	Bone formation, density, pain, swelling	Prospective study	15 patients (30 extraction sites)	Significant increase in bone density at eighth and 16th weeks reduced pain and swelling	Local application of simvastatin induces bone formation in extraction sockets
Saka-Harran et al. [37]	2022	Different doses of statins	Oral squamous cell carcinoma	Statin use, head and neck cancer risk	Hospital-based case-control study	101 cases, 101 controls	No association between prior statin use and head and neck cancer risk	Statins do not have a beneficial effect on head and neck cancer risk
Sezavar et al. [38]	2018	Simvastatin 20 mg	Healing of extraction socket	Application of simvastatin in alveolar ridge preservation	Split-mouth study	10 dental sockets	The simvastatin group showed higher percentages of vital bone, amorphous, and trabecular bone, but no significant difference compared to the control group.	Simvastatin may improve the quality of osteogenesis in the jaw bone, but further studies are necessary.
Shah et al. [39]	2015	Statin (not specified)	Bone regeneration	Effects of statins on bone regeneration, bone turnover, and regeneration via effects on cell types	Review article	N/A	Statins have pleiotropic effects, including anti-inflammatory and antimicrobial properties, and can affect bone turnover and regeneration	Statins are promising for bone regeneration and tissue engineering
Spoerl et al. [40]	2023	Statin (not specified)	OSCC patients (overall survival and recurrence-free survival)	Prognostic role of statins in OSCC	Retrospective cohort study	602	Statin use correlated with improved OAS and RFS in OSCC patients	Statin use may improve oncological outcomes in OSCC patients, but prospective clinical trials are needed
Tahamtan et al. [2]	2020	Statin (not specified)	Different aspects of dental and oral health	Effects of statins on dental and oral health	Literature review	N/A	Statins possess remarkable effects on various aspects of dental and oral health, including periodontitis, alveolar bone loss, osseointegration, and tissue healing.	Statins can be considered as novel therapeutic agents to improve dental and oral health.
Wu et al. [41]	2008	Simvastatin	Alveolar bone remodelling	Residual ridge resorption, bone turnover, bone formation, bone mineral density	Randomized controlled trial	60 male Wistar rats (30 in each group)	Significant increase in relative height of residual alveolar ridge, bone mineral density, and bone formation rate and quality in simvastatin group compared to the control group at various time points	Local application of simvastatin effectively preserves residual alveolar bone by promoting bone formation in extraction sockets
Wuster et al. [42]	2023	Rosuvastatin, simvastatin, fluvastatin, pravastatin, lovastatin, atorvastatin	Head and neck carcinoma	Influence of statin medication on overall survival of head and neck cancer patients	Retrospective clinical data analysis	48,626	Statin medication was associated with significantly improved five-year survival in head and neck cancer patients	Statin use may improve survival outcomes in head and neck cancer patients, but retrospective study design limits conclusions
Xu et al. [43]	2020	Six concentrations (0, 0.01, 0.05, 0.1, 0.5, 1)	Tooth augmentation during orthodontic tooth movement	Effects of simvastatin on orthodontic tooth movement	In vitro and in vivo study	Rat periodontal ligament cells (PDLCs)	Simvastatin triggered osteogenic differentiation of PDLCs, attenuated inflammation, and decreased osteoclastogenesis.	Simvastatin can promote bone formation and attenuate inflammation during orthodontic tooth movement.
Yaghobee et al. [44]	2020	Bovine bone material + simvastatin (1.6 gm )	Augmentation of the maxillary sinus	Efficacy of simvastatin in maxillary sinus augmentation	Randomized clinical trial with a split-mouth design	24 maxillary sinuses in 12 patients	No significant differences in newly formed bone and residual particles between the simvastatin and control groups.	Simvastatin may not have a significant positive effect on maxillary sinus augmentation.

The results show that statins have promising antiviral and antibacterial qualities, particularly against periodontal diseases and SARS-CoV-2. The results of Abdulrab et al. [18] were similar. In terms of bone regeneration, statins, especially simvastatin, have been demonstrated to dramatically enhance bone density, width, and height. Ambrósio et al. [21], Baliga et al. [23], Cai et al. [24], Degala et al. [26], Fu JH et al. [4], Gouda et al. [27], Gupta et al. [28], Hayder et al. [31], Jin et al. [32], Kabra et al. [33], Miyazawa et al. [34], Park et al. [36], Spoerl et al. [40], Tahamtan et al. [2], Wu et al. [41], and Wuster et al. all came to similar conclusions.

Furthermore, statins improved clinical attachment level rise and pocket depth, demonstrating their potential as a therapeutic adjuvant in the management of periodontal disease. Cruz et al. [25] and Aljudaibi et al. [20] came to similar conclusions. These studies also looked into the effects of statin use on osteoradionecrosis, peri-implantitis, and bone loss. The findings indicated a link between statin use and implant-related bone loss. Bahrami-Hessari et al. [22] showed similar results, although Gouda et al. [27] found that non-healing sockets could recover dramatically with an increase in alveolar height.

Additionally, it was demonstrated that statins cause apoptosis, inhibit invasion and proliferation, and reduce the production of survivin. Cai et al. [24] had similar results. Given their potential in treating dental pulp cells, alveolar bone loss, and chronic periodontitis, statins may be advantageous for oral health. Gupta S. et al. [29], Harsha et al. [30], Jin et al. [32], Kabra et al. [33], Noronha et al. [35], Saka-Harran et al. [37], Sezavar et al. [38], Shah et al. [39], Xu et al. [43], and Yaghobee et al. [44] all came to similar conclusions.

The effect of statins on the risk of head and neck cancer was also examined in the studies; the findings revealed no association between the disease's risk and previous statin use. Sezavar et al. reported similar results [38]. On the other hand, statin use was linked to improved overall and recurrence-free survival in patients with oral squamous cell carcinoma. Saifi et al. [3] obtained similar results.

Discussion

The LDL-cholesterol levels and liver cholesterol production can both be safely and effectively controlled with the statin drug family, which is a safe and effective treatment for arteriosclerotic cardiovascular disease. In addition to their strong ability to lower cholesterol, which lowers mortality and cardiovascular risk, statins are said to have a number of positive health impacts on people [1-3]. Improved endothelial function, and anti-inflammatory, antioxidant, immunomodulatory, and anti-thrombotic actions are some of these pleiotropic benefits. More and more research in recent years indicates that statins may benefit dental and oral health via many pathways [2].

Statins, particularly simvastatin, were observed to have a major impact on improving oral health and bone regeneration across the articles that we analyzed in this review. Our assessment is consistent with the study conducted by Abdulrab et al. [18], which demonstrated that topical statins may reduce the incidence of SARS-CoV-2 transmission in dentistry settings. In a similar vein, simvastatin may not retain alveolar bone height, according to the study conducted by Abu Sheehah et al. [19]. This is in line with the results of studies conducted by Aljudaibi et al. [20] and Ambrósio et al. [21].

Additionally, our research showed that the results of Baliga et al. [23] and Cai et al. [24] complement the study by Bahrami-Hessari et al. [22], which found that statins may have a favorable effect on peri-implant bone loss. In addition, Cruz et al.'s study [25] supported the findings of Degala et al. [26] and Fu et al. [4] by indicating that statins might be a promising addition to periodontal therapy.

Furthermore, we discovered that Gouda et al.'s study [27] supported the findings of Gupta et al. [28] and Gupta et al. [29] by concluding that simvastatin is safe and has the potential capacity to induce osteoinduction. Furthermore, research by Hayder et al. [31] and Jin et al. [32] supports the work by Harsha et al. [30], which hypothesized that simvastatin would help oral bone and cartilage recover.

In line with the findings of Miyazawa et al. [34] and Noronha et al. [35], our research also showed that the study by Kabra et al. [33] discovered that simvastatin is more effective in stimulating bone regeneration compared to hydroxyapatite and platelet-rich fibrin. Furthermore, although additional research is required, the Park et al. study [36] revealed that statins may enhance peri-implant bone repair.

We also contrasted the results of Saka-Harran et al. [37] and Saifi et al. [3], which revealed that statins significantly improve bone regeneration and dental health. Additionally, our data showed that simvastatin may enhance the quality of osteogenesis in the jaw bone, as reported by Sezavar et al. [38]. These findings are corroborated by those of Shah et al. [39] and Spoerl et al. [40].

Additionally, we noted that Tahamtan et al.'s work [2] supported the findings of Wu et al. [41] and Wuster et al. [42] by concluding that statins show promise for bone regeneration and tissue engineering. Furthermore, simvastatin may help to stimulate bone formation and reduce inflammation during orthodontic tooth movement, according to Xu et al.'s study [43] and Yaghobee et al.'s findings [44].

A considerable proportion of the populace is afflicted with coronary heart disease (CHD), a common medical condition. One of the leading causes of death in the US is CHD. For those 65 years of age and older, it is the main cause of stroke or death. Among young people (those between the ages of 20 and 45), the prevalence of CHD is almost 65% [1-3]. Hyperlipidaemia, also known as hypercholesterolaemia, is the most modifiable risk factor for CHD and is very common in the US population. This is the reason statins, or drugs that lower cholesterol, are often prescribed. Their main method of action is inhibiting HMG-CoA reductase activity, which impedes the synthesis of cholesterol. New evidence suggests that statins may affect bone metabolism [3, 45].

Although many strategies have been proposed, there is currently no evidence to support an optimal strategy or biomaterial for the maintenance of alveolar ridges [28-29]. Therefore, more information on biomaterials and procedures is required to increase the processes' predictability and reproducibility.

One advantage of applying osteoinductive drugs locally is the reduction of toxicity and side effects. Two further advantages are improved patient compliance and postponed medication release [2, 7]. Compared to systemic statin use, local osteoinduction appears to be 50 times more linked with statin use [9].

A semi-synthetic equivalent of lovastatin, simvastatin, has been demonstrated to enhance bone metabolism. Elevated bone mineral density (BMD) was found to be associated with increases in osteocalcin, bone alkaline phosphatase, and the type I collagen C-terminal telopeptide [46]. Women with type II diabetes who had experienced menopause also showed comparable results [46]. Hydrophilic statins had no discernible effects on serum osteocalcin, alkaline phosphatase, atorvastatin, fluvastatin, or the C-terminal telopeptide of type I collagen [47]. It was therefore anticipated that lipophilic statins would influence bone remodeling more than other statins [3].

Nyan et al. [48] provided an explanation for the simvastatin burst release event that occurred from the graft particles on the first day, which was followed by a delayed release. This is advantageous since the proper dosage of the drug promotes BMP-2 production in surrounding cells without inducing an inflammatory reaction. Because of its regenerative properties, simvastatin may be used as a medication for soft tissue regeneration and repair [49], and due to the fact that it can reduce inflammatory mediators and accelerate soft tissue regeneration, this is especially advantageous in cases of periapical infections and extraction sockets [50-51].

We admit that the accuracy of our conclusions may have been hampered by the variability of the included studies. The generalisability of our findings may have been impacted by biases and confounding variables introduced by the variations in study designs, sample sizes, and materials used. Moreover, our review was limited to publications written in English, which might have excluded pertinent works written in other languages. Furthermore, because of the precise keywords and databases we utilized, it's possible that our search method overlooked pertinent studies.

In light of our findings, we suggest that more rigorous study designs, bigger sample sizes, and standardized materials be used in future research examining the use of statins in oral and maxillofacial surgery. To further comprehend the therapeutic potential of statins, we further recommend that researchers investigate the underlying processes of these drugs' effects on periodontal disease and bone regeneration. Furthermore, we suggest that physicians think about using statins as an additional treatment for patients having oral and maxillofacial surgery or periodontal disease.

## Conclusions

The cumulative evidence analyzed in our review indicates that statins have a significant effect on enhancing bone regeneration, a crucial factor in the field of oral and maxillofacial surgery, where achieving the best possible tissue repair is vital for achieving favorable results. Moreover, statins have been shown to have antiviral and antibacterial characteristics, rendering them a useful supplementary therapy in the management of periodontal disease, a widespread phenomenon that presents substantial obstacles to oral health. The literature analysis emphasizes the possible advantages of statins as a supplementary treatment in the management of periodontal disease. It highlights their ability to regulate the biological response of the host, decrease inflammation, and stimulate the regeneration of tissues. Although the current body of evidence strongly supports the use of statins in oral and maxillofacial surgery, more research is required to completely understand the mechanisms responsible for their effects and to determine their effectiveness in different clinical situations. This will ultimately help to develop evidence-based recommendations for their integration into daily clinical practice.
